# Risk of Ischemic Heart Disease and Stroke in Prostate Cancer Survivors: A Nationwide Study in South Korea

**DOI:** 10.1038/s41598-020-67029-y

**Published:** 2020-06-25

**Authors:** Dong Wook Shin, Kyungdo Han, Hyun Sik Park, Seung-Pyo Lee, Sang Hyun Park, Jinsung Park

**Affiliations:** 1 Supportive Care Center/Department of Family Medicine, Samsung Medical Center, Sungkyunkwan University School of Medicine, Seoul, Korea; 2 Department of Digital Health, SAIHST, Sungkyunkwan University, Seoul, Korea; 3 Department of Statistics and Actuarial Science, Soongsil University, Seoul, Korea; 4 Department of Urology, Eulji University Hospital, Eulji University School of Medicine, Daejeon, Korea; 5 Division of Cardiology, Department of Internal Medicine, Seoul National University Hospital, Seoul National University College of Medicine, Seoul, Korea; 6 Department of Medical Statistics, Catholic University of Korea, Seoul, Korea

**Keywords:** Urology, Prostate

## Abstract

In this study using national health insurance data, we investigated the risk of ischemic heart disease (IHD) and stroke among prostate cancer (PC) survivors compared with the general population, as well as the risk of cardiovascular disease (CVD) according to primary treatment. A total of 48,298 PC patients diagnosed from 2007 to 2013 were included and matched to non-cancer controls. Compared to the general population, PC survivors had a slightly lower risk of IHD (adjusted hazard ratio [aHR] = 0.89, 95% confidence interval [CI] 0.83–0.96) or stroke (aHR 0.90, 95% CI 0.87–0.95). Especially, survivors who underwent surgery had lower risks of IHD (aHR 0.70, 95% CI 0.61–0.80) or stroke (aHR 0.73, 95% CI 0.67–0.81). Compared to survivors in the active surveillance/watchful waiting group, the androgen deprivation therapy (ADT) group had a significantly greater risk of stroke (aHR 1.16, 95% CI 1.02–1.32), but the IHD risk was not significantly elevated (aHR 1.06, 95% CI 0.88–1.29). In conclusion, PC survivors had a slightly lower risk of CVD compared to the general population, which was attributable to self-selection for PSA screening, specifically in the surgery-only group. CVD risk was dependent on treatment received, and attention should be given to patients who receive ADT.

## Introduction

Cancer survivor is defined as any person diagnosed with cancer, from the time of diagnosis until his or her death. With improved survival and the increased number of prostate cancer (PC) survivors^1^, management of comorbidities has become increasingly important for this population^2^. Cardiovascular disease (CVD) is reported to be the main cause of mortality in PC survivors in the US, comprising 20% of overall mortality, and surpassing mortality from PC and second primary malignancies^3^. In a Korean cohort study, CVD was responsible for 29.1% of non-PC mortality in long-term PC survivors^4^.

Many studies have analyzed the risk of CVD in PC patients treated with androgen deprivation therapy (ADT)^5–17^, but it is not certain whether PC survivors have a greater risk of CVD compared to the general population. To date, only a few studies have compared the CVD risk between PC survivors and the general population^18–20^, but the results were inconsistent: a Swedish study suggested elevated risk^18^, a UK study reported similar risk^19^, and a US study demonstrated lower risk^20^. Among these studies, only the Swedish study analyzed the CVD risk according to treatment modality (i.e., surveillance, curative treatment, endocrine therapy)^18^. Thus, the risk of CVD among PC survivors who underwent different types of treatment has not been assessed definitively. In addition, there are no reports from Asian countries, where practice patterns may differ from Western countries^21^. To address these limitations, we used a Korean national healthcare database to investigate the CVD risk among PC survivors compared with general population controls, as well as the risk of CVD according to primary treatment.

## Methods

### Data Source: Korean National Health Insurance Service (NHIS) database

The Korean National Health Insurance Service (KNHIS) is a single-government payer which provides a mandatory public health insurance program to virtually the entire Korean population (around 97%). The remaining 3% of the population with the lowest income is covered by the Medical Aid program financed from general taxes, but the administration for these people is also covered by the NHIS. Medical services are mainly provided by private providers, and they are reimbursed from the KNHS for their service provision.

The KNHIS also provides a free biennial cardiovascular health screening program to all Koreans over 40 years of age and to those who are employed regardless of age^22^. This program consists of (1) anthropometric measurements (height, weight, blood pressure, etc.), (2) health behavior assessment (smoking, alcohol intake, etc.), and (3) laboratory tests (blood glucoses, lipid level).

Therefore, the KNHIS database^22,23^ contains all the information necessary for reimbursement of each medical service, and includes (1) beneficiary information (age, sex, residential area, and income status); (2) medical claims information (disease codes based on international classification of disease [ICD] version 10, outpatient clinic visits and inpatient admissions, diagnostic tests, procedures and other medical treatments performed, prescription, and incurred costs); and (3) national health screening data.

### Study population

We selected a total of 81,445 patients newly diagnosed with PC (ICD-10 code C61) from January 1, 2007 to December 31, 2013. We excluded patients who (1) were less than 19 years old (N = 16); (2) had been diagnosed with other cancers (C00-C97 except C61, N = 22,305) prior to their PC diagnosis; (3) had a previous history of ischemic heart disease (IHD) (I20-I25) or stroke (I63 or I64) prior to their PC diagnosis (N = 5,141); and (4) had follow-up of less than one year from the date of PC diagnosis (N = 5,955). In total, 48,298 PC patients were included in the final analyses. Among those, 29,365 patients had participated in the Korean cardiovascular health screening program in the year prior to their PC diagnosis and thus had information addressing baseline smoking status and BMI: these patients comprised the screening subset population.

**Figure 1:**
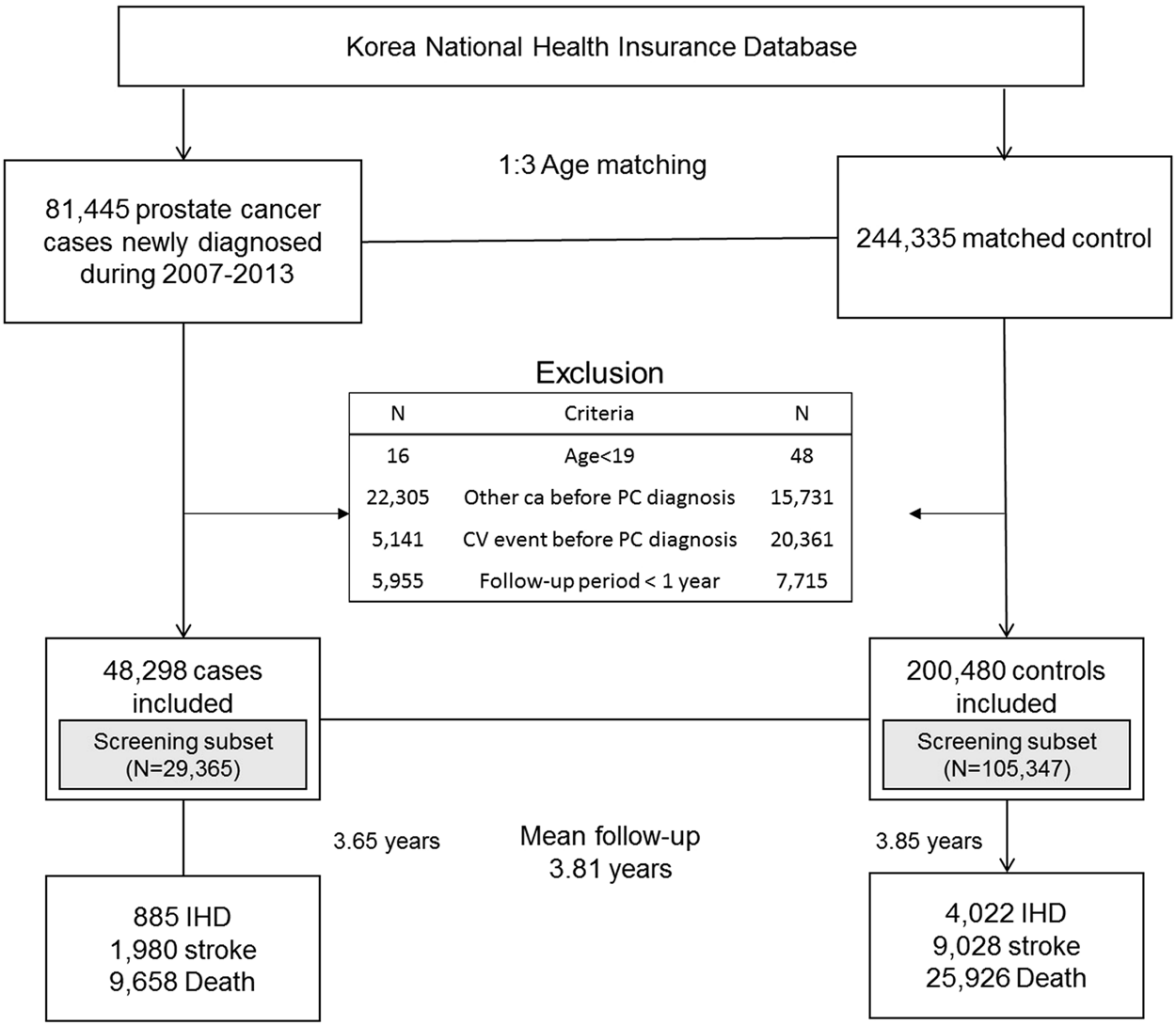
Selection Scheme for the Study Population.

For selection of the control group, we used 1:3 age matching. Matching was performed serially year by year such that PC patients diagnosed at a specific year were matched on the basis of age and sex to control subjects who were alive during the same year. Control subjects were assigned an index date corresponding to the date of PC diagnosis of the matched patients. The same exclusion criteria used to screen the study patients were applied to select the matched control subjects: (1) <19 years (N = 48); (2) previous cancer history (N = 15,731); (3) previous IHD or stroke (N = 20,361); and (4) had follow-up of <1 year from index date (N = 7,715). A total of 200,480 matched controls were included in the final analyses, and 105,347 of these comprised the screening subset population. The study population selection scheme is illustrated in Fig. 1.

### Study outcomes and follow-up

The endpoints of the study were newly-diagnosed IHD, stroke, or death. IHD was defined as the recording of ICD-10 codes I20-I25 during hospitalization, and stroke was defined as the recording of ICD-10 codes I63 or I64 during hospitalization with claims for brain magnetic resonance imaging or brain computerized tomography. The study population was followed from one year after the PC diagnosis or index date to the date of cardiovascular event, death, or until December 31, 2016, whichever came first.

### Statistical analysis

Descriptive statistics were used to determine the basic characteristics of the PC survivors and matched controls. Cox proportional hazards regression analyses were used to determine the relative risk for each study outcome. The proportional hazard assumption was checked by a log-log plot to ensure the validity of the Cox regression model. Univariate analyses were presented as Model 1. An age-adjusted model was presented as Model 2. A multivariate model adjusted for age, income, Charlson comorbidity index, hypertension, diabetes, and dyslipidemia was presented as Model 3. The screening subset was further adjusted for smoking status and BMI, blood glucose, systolic blood pressure, and total cholesterol (Model 4).

As PC patients are more likely to die of PC than matched controls, and PC death can be a competing event for the incidence of CHD and stroke, we also performed a competing risk regression analysis by taking into account excess mortality in PC survivors. A semiparametric proportional hazards model by Fine and Gray was used, and the risk was presented as subdistribution hazard ratio (SHR) and 95% confidence interval (CI).

To examine the different risk of IHD and stroke among PC survivors with different treatments compared to the matched control group, analyses by treatment type were performed: (1) active surveillance/watchful waiting (AS/WW); (2) surgery; (3) surgery + ADT; (4) radiotherapy (RT) + ADT; (5) ADT only; and (6) RT only. The AS/WW group was defined as patients who did not receive active treatment after PC diagnosis. ADT included both surgical (orchiectomy) and medical castration, which comprised administration of luteinizing hormone-releasing hormone (LHRH) agonists, antiandrogen monotherapy, combined androgen blockade, and/or estrogen. The duration of ADT use was defined operationally as the interval from the date of initial prescription to the date of last prescription plus one month. All statistical analyses were performed using SAS version 9.1 (SAS institute, Cary, NC) and *P* values <0.05 were considered significant.

### Ethics statement

This study was approved by the Institutional Review Board of Eulji University Hospital (No. 2018-09-001). The requirement for informed consent was waived, as this study used de-identified data from administrative database. All methods were carried out in accordance with relevant guidelines and regulations.

## Results

### Characteristics of the Study Population

At baseline, PC survivors were more likely to be within higher income quartiles (36.6% vs. 29.4%) and living in urban areas (46.9% vs. 44.4%), and had more hypertension (49.3% vs. 40.0%), diabetes mellitus (17.6% vs. 15.3%), dyslipidemia (21.4% vs. 13.3%), and used more aspirin (6.3% vs. 5.0%) and statins (22.7% vs. 16.7%) than matched control subjects (P < 0.001). Among PC survivors undergoing ADT, the mean duration of ADT was 3.0 years (SD 2.2).

The screening subset included 29,365 PC survivors and 105,347 non-cancer control subjects. PC survivors had a lower current smoking rate (21.2% vs. 27.1%), and higher BMI (P < 0.001) **(**Table 1**)**.

**Table 1:** Baseline characteristics of the study participants.

	Study population (all)		p	Screening subset		p
	Prostate cancer population	Matched controls		Prostate cancer population	Matched controls	
n (%)	48,298	200,480		29,365	105,347	
Age ± SD	68.4 ± 8.7	67.6 ± 9.4	<0.001	67.8 ± 8.2	66.9 ± 8.8	<0.0001
Income level			<0.001			<0.0001
Rank 1-5 (lowest)	10,773 (22.3)	54,515 (27.2)		6,020 (20.5)	25,364 (24.08)	
Rank 6-10	8,425 (17.4)	39,261 (19.6)		5,412 (18.4)	21,666 (20.6)	
Rank 11-15	11,408 (23.6)	47,780 (23.8)		7,203 (24.5)	26,273 (24.9)	
Rank 16-20 (highest)	17,692 (36.6)	58,924 (29.4)		10,730 (36.5)	32,044 (30.4)	
Place of residence, urban	22,642 (46.9)	89,064 (44.4)	<0.001	13,334 (45.4)	45,824 (43.5)	<0.0001
Hypertension	23,816 (49.3)	80,194 (40.0)	<0.0001	14,305 (48.7)	43,613 (41.4)	<0.0001
Diabetes Mellitus	8,492 (17.6)	30,642 (15.3)	<0.0001	4,829 (16.4)	16,039 (15.2)	<0.0001
Dyslipidemia	10,342 (21.4)	26,700 (13.3)	<0.0001	6,306 (21.5)	15,265 (14.5)	<0.0001
Charlson comorbidity index	1.6 ± 1.8	1.3 ± 1.6	<0.0001	1.6 ± 1.7	1.3 ± 1.6	<0.0001
Use of aspirin	3,024 (6.3)	10,050 (5.0)	<0.0001	1,664 (5.7)	5,082 (4.8)	<0.0001
Use of statins	10,945 (22.7)	33,370 (16.7)	<0.0001	6,632 (22.6)	19,047 (18.1)	<0.0001
Smoking status						<0.0001
None				14,005 (47.7)	46,920 (44.5)	
Past				9,127 (31.1)	29,844 (28.3)	
Current				6,233 (21.2)	28,583 (27.1)	
Body mass index						<0.0001
<18.5				777(2.7)	3,634 (3.5)	
18.5-23				9,984 (34)	38,551 (36.6)	
23-25				8,521 (29.0)	29,128 (27.7)	
25-30				9,499 (32.4)	31,976 (30.4)	
≥30				584 (2.0)	2,058 (2.0)	

P values were calculated by t-test for continuous variables, and chi-square test for categorical variables.

### CVD Risk in PC Survivors Compared to Matched Controls

The mean durations of follow-up after 1 year of PC diagnosis or index date were 3.81, 3.65 and 3.85 years for all subjects, PC survivors, and matched control subjects, respectively. In conventional Cox regression analyses, PC survivors were found to have a slightly lower risk of IHD (adjusted hazard ratio [aHR] = 0.89, 95% confidence interval [CI] 0.83–0.96) and stroke (aHR 0.90, 95% CI 0.86–0.95), while their overall risk of death was greater (aHR 1.61, 95% CI: 1.57–1.64) than that of matched controls. When further adjusted for smoking, BMI, blood glucose, blood pressure, and total cholesterol in the screening subset, the overall pattern was generally similar, but the aHRs for IHD (aHR 0.98, 95% CI 0.88–1.08) or stroke incidence (aHR 0.98, 95% CI 0.91–1.05) were not statistically significant **(**Table 2**)**. Competing risk regression analysis also produced similar results showing slightly lower risk of IHD and stroke in PC survivors **(**Supplementary Table 1**)**.

**Table 2:** Risk of ischemic heart disease, stroke, and death in prostate cancer patients compared to the matched comparison group.

	N	Event	Person-years	IR (per 1000)	Model 1	Model 2	Model 3	Model 4
**All participants**								
**Ischemic heart disease**								
Control	200,480	4,022	764,737.6	5.3	1(Ref.)	1(Ref.)	1(Ref.)	
Case	48,298	885	174,673.7	5.1	0.96 (0.90, 1.04)	0.94 (0.87, 1.01)	0.89 (0.82, 0.95)	
**Stroke**								
Control	200,480	9,028	754,976.4	12.0	1(Ref.)	1(Ref.)	1(Ref.)	
Case	48,298	1,980	172,703.5	11.5	0.96 (0.91, 1.01)	0.93 (0.89, 0.98)	0.90 (0.86, 0.95)	
**Death**								
Control	200,480	25,926	771,765.1	33.6	1(Ref.)	1(Ref.)	1(Ref.)	
Case	48,298	9,658	176,054.8	54.9	1.64 (1.60, 1.68)	1.60 (1.56, 1.63)	1.61 (1.57, 1.64)	
**Screening subset**								
**Ischemic heart disease**								
Control	105,347	1,766	390,160.3	4.5	1(Ref.)	1(Ref.)	1(Ref.)	1(Ref.)
Case	29,365	477	103,946.2	4.6	1.02 (0.92, 1.12)	0.98 (0.89, 1.09)	0.95 (0.85, 1.05)	0.98 (0.88, 1.08)
**Stroke**								
Control	105,347	4,002	385,781.1	10.4	1(Ref.)	1(Ref.)	1(Ref.)	1(Ref.)
Case	29,365	1,075	102,905.3	10.4	1.01 (0.94, 1.08)	0.97 (0.91, 1.04)	0.95 (0.89, 1.01)	0.98 (0.91, 1.05)
**Death**								
Control	105,347	9,202	393,364.8	23.4	1(Ref.)	1(Ref.)	1(Ref.)	1(Ref.)
Case	29,365	4,611	104,694.0	44.0	1.89 (1.83, 1.96)	1.81 (1.75, 1.87)	1.82 (1.76, 1.89)	1.90 (1.84, 1.97)

IR: incidence rate.

Model 1: crude model.

Model 2: adjusted for age.

Model 3: adjusted for age, income, Charlson comorbidity index, diabetes mellitus, hypertension, and dyslipidemia.

Model 4: adjusted for age, income, Charlson comorbidity index, diabetes mellitus, hypertension, dyslipidemia, smoking status, BMI, blood glucose, systolic blood pressure, and total cholesterol.

### CVD Risk in PC survivors by treatment modalities compared to matched control

The AS/WW group was generally found to have a similar risk of CVD to matched non-cancer controls. In a multivariate model, survivors who received only surgery had lower risks of IHD (aHR 0.69, 95% 0.60–0.80) or stroke (aHR 0.73, 95% CI 0.66–0.80). The surgery+ ADT group had a marginally lower risk of IHD (aHR 0.82, 95% CI 0.66–1.02), and significantly lower risk of stroke (aHR 0.78, 95% CI 0.67–0.91). The RT + ADT group had lower risks of IHD (aHR 0.58, 95% CI 0.31–1.08) or stroke (aHR 0.83, 95% CI 0.58–1.18), but the differences were not statistically significant. The results for the ADT-only or RT-only groups were not significantly different from the control group **(**Table 3**)**. Subgroup analyses with the screening subset showed similar results indicating a lower risk of IHD (aHR: 0.81, 95% CI: 0.68–0.97) or stroke (aHR: 0.80, 95% CI: 0.71–0.91) in the surgery only group compared to controls, but the difference was smaller than in the total population **(**Supplementary Table 2**)**.

**Table 3:** Risk of ischemic heart disease, stroke, and death in prostate cancer patients by treatment modality compared to the matched comparison group: all participant**s**.

	N	Event	Duration	IR (per 1000)	Model 1	Model 2	Model 3
**Ischemic heart disease**							
Control	200,480	4,022	764,737.6	5.3	1 (Ref.)	1 (Ref.)	1 (Ref.)
AS/WW	6,964	155	28,791.0	5.4	1.02 (0.87, 1.20)	1.05 (0.89, 1.23)	0.98 (0.83, 1.15)
Surgery	17,425	208	64,240.6	3.2	0.62 (0.54, 0.71)	0.73 (0.64, 0.84)	0.69 (0.60, 0.80)
Surgery + ADT	5,573	85	20,900.6	4.1	0.77 (0.62, 0.96)	0.87 (0.70, 1.08)	0.82 (0.66, 1.02)
RT + ADT	1,285	10	2,806.0	3.6	0.68 (0.36, 1.26)	0.62 (0.33, 1.15)	0.58 (0.31, 1.08)
ADT	16,624	420	57,052.1	7.4	1.40 (1.27, 1.55)	1.08 (0.97, 1.19)	1.02 (0.92, 1.12)
RT	427	7	883.4	7.9	1.50 (0.72, 3.16)	1.31 (0.62, 2.74)	1.16 (0.55, 2.44)
**Stroke**							
Control	200,480	9,028	754,976.4	12.0	1 (Ref.)	1 (Ref.)	1 (Ref.)
AS/WW	6,964	324	28,418.0	11.4	0.95 (0.85, 1.06)	0.98 (0.88, 1.09)	0.93 (0.84, 1.04)
Surgery	17,425	462	63,763.2	7.2	0.61 (0.55, 0.67)	0.75 (0.68, 0.82)	0.73 (0.66, 0.80)
Surgery + ADT	5,573	173	20,754.9	8.3	0.70 (0.6, 0.81)	0.81 (0.70, 0.94)	0.78 (0.67, 0.91)
RT + ADT	1,285	31	2,781.7	11.1	0.94 (0.66, 1.34)	0.85 (0.60, 1.21)	0.83 (0.58, 1.18)
ADT	16,624	978	56,110.4	17.4	1.46 (1.36, 1.56)	1.07 (1.01, 1.15)	1.03 (0.97, 1.10)
RT	427	12	875.2	13.7	1.16 (0.66, 2.04)	0.98 (0.56, 1.73)	0.91 (0.52, 1.61)
**Death**							
Control	200,480	25,926	771,765.1	33.6	1 (Ref.)	1 (Ref.)	1 (Ref.)
AS/WW	6,964	1,638	29,043.2	56.4	1.66 (1.58, 1.74)	1.73 (1.64, 1.82)	1.73 (1.64, 1.82)
Surgery	17,425	1,100	64,603.9	17.0	0.51 (0.48, 0.54)	0.73 (0.68, 0.77)	0.75 (0.70, 0.79)
Surgery + ADT	5,573	539	21,059.2	25.6	0.77 (0.70, 0.83)	1.00 (0.92, 1.09)	1.01 (0.93, 1.10)
RT + ADT	1,285	130	2,812.3	46.2	1.45 (1.22, 1.72)	1.31 (1.11, 1.56)	1.36 (1.15, 1.62)
ADT	16,624	6,192	57,647.1	107.4	3.21 (3.12, 3.30)	2.12 (2.06, 2.18)	2.11 (2.05, 2.17)
RT	427	59	889.1	66.4	2.08 (1.61, 2.69)	1.73 (1.34, 2.23)	1.78 (1.38, 2.30)

IR: incidence rate.

Model 1: crude model.

Model 2: adjusted for age.

Model 3: adjusted for age, income, Charlson comorbidity index, diabetes mellitus, hypertension, and dyslipidemia.

### CVD Risk in PC survivors by treatment modalities compared to the AS/WW group

Compared to survivors in the AS/WW group, survivors who received only surgery were found to have lower risks of IHD (aHR 0.69, 95% CI 0.56–0.85) or stroke (aHR 0.75, 95% CI 0.65–0.87); similarly, the surgery+ ADT group also had a significantly lower risk of stroke (aHR 0.81, 95% CI 0.68–0.98), but had only a marginally lower risk of IHD (aHR 0.82, 95% CI 0.63–1.08). The RT + ADT group had a lower risk of IHD (aHR 0.57, 95% CI 0.30–1.09) and stroke (aHR 0.88, 95% CI 0.61–1.28), but these findings were not statistically significant.

**Figure 2:**
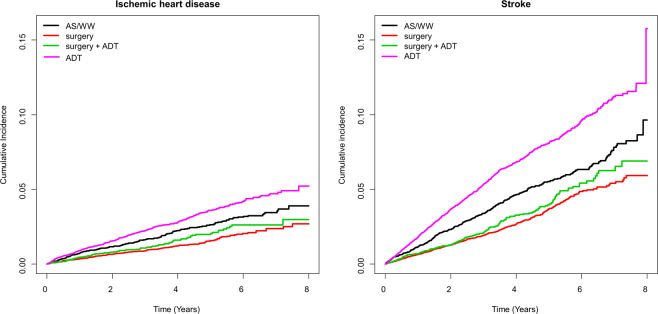
The Kaplan-Meier curves^*^ for risk of ischemic heart disease and stroke according to primary treatments in prostate cancer survivors. ^*^Survivors undergoing radiation therapy (RT) and RT + ADT were not shown in the curves due to the small number of patients. Abbreviations: AS/WW: Active surveillance/watchful waiting; ADT: Androgen Deprivation Therapy.

The ADT-only group had a significantly greater risk of stroke (aHR 1.16, 95% CI 1.02–1.32), but the IHD risk was not significantly elevated (aHR 1.07, 95% CI 0.88–1.29). The RT-only group was small, and this group’s results were not different from the AS/WW group **(**Table 4**)**. Subgroup analyses with the screening group subset also showed similar estimates indicating a lower risk of IHD (aHR: 0.78, 95% CI: 0.58–1.05) or stroke (aHR: 0.74, 95% CI: 0.61–0.91) in the surgery only group compared to the AS/WW group **(**Supplementary Table 3**)**. Kaplan-Meier curves showing the incidence of CVD over time are shown in Fig. 2.

**Table 4:** Risk of ischemic heart disease, stroke, and death in prostate cancer patients by treatment modality compared to the AS/WW group: all participants.

	N	Event	Person-year	IR(per 1000)	Model 1	Model 2	Model 3
**Ischemic heart disease**							
AS/WW	6,964	155	28,791.0	5.4	1 (Ref.)	1 (Ref.)	1 (Ref.)
Surgery	17,425	208	64,240.6	3.2	0.60 (0.49, 0.74)	0.68 (0.55, 0.84)	0.69 (0.56, 0.85)
Surgery + ADT	5,573	85	20,900.6	4.1	0.75 (0.58, 0.98)	0.81 (0.62, 1.06)	0.82 (0.63, 1.08)
RT + ADT	1,285	10	2,806.0	3.6	0.64 (0.33, 1.22)	0.57 (0.30, 1.09)	0.57 (0.30, 1.09)
ADT	16,624	420	57,052.1	7.4	1.35 (1.13, 1.63)	1.05 (0.87, 1.27)	1.07 (0.88, 1.29)
RT	427	7	883.4	7.9	1.40 (0.66, 3.00)	1.21 (0.57, 2.60)	1.17 (0.55, 2.50)
**Stroke**							
AS/WW	6,964	324	28,418.0	11.4	1 (Ref.)	1 (Ref.)	1 (Ref.)
Surgery	17,425	462	63,763.2	7.2	0.64 (0.55, 0.74)	0.74 (0.64, 0.85)	0.75 (0.65, 0.87)
Surgery + ADT	5,573	173	20,754.9	8.3	0.73 (0.61, 0.88)	0.80 (0.67, 0.97)	0.81 (0.68, 0.98)
RT + ADT	1,285	31	2,781.7	11.1	0.98 (0.68, 1.43)	0.88 (0.60, 1.27)	0.88 (0.61, 1.28)
ADT	16,624	978	56,110.4	17.4	1.53 (1.35, 1.74)	1.15 (1.01, 1.31)	1.16 (1.02, 1.32)
RT	427	12	875.2	13.7	1.21 (0.68, 2.16)	1.02 (0.57, 1.82)	0.98 (0.55, 1.74)
**Death**							
AS/WW	6964	1,638	29,043.2	56.4	1 (Ref.)	1 (Ref.)	1 (Ref.)
Surgery	17,425	1,100	64,603.9	17.0	0.30 (0.28, 0.32)	0.33 (0.31, 0.36)	0.33 (0.31, 0.36)
Surgery + ADT	5,573	539	21,059.2	25.6	0.45 (0.41, 0.49)	0.48 (0.43, 0.53)	0.47 (0.43, 0.52)
RT + ADT	1,285	130	2,812.3	46.2	0.77 (0.64, 0.93)	0.70 (0.58, 0.84)	0.71 (0.60, 0.85)
ADT	16,624	6,192	57,647.1	107.4	1.87 (1.78, 1.98)	1.50 (1.42, 1.59)	1.47 (1.39, 1.56)
RT	427	59	889.1	66.4	1.1 (0.85, 1.43)	0.97 (0.74, 1.25)	0.99 (0.76, 1.29)

IR: incidence rate.

Model 1: crude model.

Model 2: adjusted for age.

Model 3: adjusted for age, income, Charlson comorbidity index, diabetes mellitus, hypertension, and dyslipidemia.

## Discussion

This large population-based cohort study with a robust comparison population found that CVD risk was slightly lower among PC survivors compared to the general population (matched controls), and the risks for IHD and stroke were different in patient groups who received different primary treatment methods. Specifically, PC survivors who underwent surgery were found to have significantly lower risk of CVD (both IHD and stroke) compared to the control group; however, the risk was similar in both groups when the analysis was confined to a screening group subset and further adjusted for BMI and smoking status. When risk of CVD was assessed by treatment modality, the ADT group was found to have similar risk to the control group, but greater risk compared to the surgery-only group. The major strengths of our study include the large sample size, use of age-matched non-cancer controls, low attrition, comparison between various treatment methods, and extensive adjustment for various sociodemographic and major cardiovascular risk factors, such as comorbidities, drug use, lifestyle, anthropometric and laboratory results, which was achieved by linkage of healthcare claims and health screening data.

A limited number of studies^18–20^ have reported inconsistent results regarding the CVD risk of PC survivors compared to the general population. A Swedish study^18^ using the National Prostate Cancer Register reported that PC survivors had a higher risk of CVD compared to the general population, but the risk varied according to the treatment method: standardized incidence ratios [SIR] of 1.22 and 1.19 for IHD and stroke, respectively, were reported for those who underwent only surveillance, and 1.06 and 0.98 for those who underwent curative treatment; and 1.32 and 1.26 for patients who received ADT. However, another UK study involving five-year PC survivors reported no difference in the risk of CVD compared to the matched controls, but this study did not have any treatment information and the authors did not identify definitive reasons for their findings^19^. A US study based on a SEER-affiliated cancer registry (using data from 2000 to 2007) reported PC survivors who survived >two years had a lower CVD risk (incidence rate ratio [IRR], 0.89 [95% CI 0.84–0.95]) compared with matched controls drawn from the general population^20^; however, treatment information was likewise not available for the study population. The conflicting results from previous studies may reflect the differences in eligibility criteria (all vs. >2-year vs. >5-year survivors) and in PC treatment and CVD management practice in different countries.

Our study showed that PC survivors had a slightly lower risk of CVD (aHR 0.89 for IHD, aHR 0.90 for stroke) compared to matched controls drawn from the general population, but when limited to a screening subset, the risks were similar (aHR 0.98 for both IHD and stroke). In Korea, PSA screening is not provided by government and is paid for by the patients themselves^24^. Therefore, PC survivors are those who have better access to that healthcare program, as evidenced by higher income ranks in study subjects compared with the general population. In addition, Koreans who elected to receive health screening were also more likely to engage in healthier behavior (e.g., not smoking) and received more attentive preventive treatment (e.g., antihypertensive agents, statins, and aspirin)^22^. Therefore, when we homogenized the study population by confining it to screening participants, the observed difference between PC survivors and matched controls was diminished. In addition, we postulate that the discrepancy between the findings of previous studies can be understood in the context of the availability of PSA screening: PSA screening was not commonly performed in Sweden^18^, but it is commonly practiced in the US^20^. Therefore, our findings appear to be similar to previous findings in the US^20^.

Similar to the findings in patients who participated in health screening, PC survivors who underwent surgery without other therapy had markedly lower CV risk (aHR 0.70 for IHD and 0.73 for stroke) than the general population. PC survivors who were treated only with surgery were generally more likely to undergo regular health screening than the control population, and such health-seeking behavior patterns decrease the risk of developing CVD, as shown in our previous study^22^. Similar findings were noted in the US study^20^: PC survivors with early-stage (SEER stage I/II) cancers and who were likely to be managed by curative surgery had a lower risk of CVD (IRR 0.89, 95% CI 0.84–0.95), while the CVD risk was not different in stage IV patients (IRR 1.03, 95% CI 0.78–1.35), who were managed primarily by ADT. The authors of this study^20^ suggested that PC patients with early-stage disease are more likely to undergo routine screening for early diagnosis of PC and hence more likely to participate in the health care system for preventive care.

The most controversial issue regarding the CVD risk in PC survivors is ADT. In our study, compared to general population or AS/WW group, PC survivors receiving ADT only generally had similar risks of IHD (aHR 1.01 and 1.06, respectively) or stroke (aHR 1.03 and 1.16, respectively). However, compared to the surgery-only group, the ADT group had markedly greater risk of IHD and stroke. While many studies have shown that ADT is associated with CVD^5–14^, other studies^15–17^ have demonstrated conflicting results, which were attributed to differences in the prior history of CV events or type and duration of ADT. A meta-analysis using randomized controlled trials (RCTs) suggested no increased risk of fatal CVD associated with ADT^17^, but pooled analyses of observational studies showed consistent evidence of increased risk of CVD, regardless of the type of ADT therapy^6^. This discrepancy was mainly explained by differences in outcome assessment (only fatal vs. fatal and non-fatal) and study population (e.g., RCTs usually excluded older patients with multiple comorbidities)^6^. Our study results are in line with the pooled analyses of observational studies^6^, and suggest the possibility of increased risk in PC patient receiving ADT. On the other hand, the similar risks of CVD of the ADT group compared to the general population and the AS/WW group can be explained by the inclusion criteria of our study. Previous studies suggested that increased CVD risk following ADT is marked in patients with a prior history of CVD^18^ and a greater number of risk factors for CVD^25^, and the increased CVD risk in men receiving ADT is mainly evident within six months after starting ADT^25^. Meanwhile, participants in our study were limited to ≥1-year PC survivors and patients with CVD at baseline were excluded from our study population, which may explain why we found no markedly increased risk of CVD in our patients as compared to the general population or AS/WW group. Interestingly, the surgery + ADT group had a higher risk of CVD compared to the surgery-only group, probably due to the effect of ADT, consistent with the results of a prior study^5^.

In general, RT seemed not to be associated with CVD risk, although the number of subjects may have been too small to evaluate any effect. Unfortunately, owing to the procedure codes used by the KNHIS, we could not determine whether RT was performed for curative or palliative purposes. However, RT is rarely performed in Korea as a primary treatment^21^, and significant proportions of patients undergoing RT (RT only or RT + ADT) may have been those who received palliative treatment. Thus, we postulate that in our study PC survivors undergoing RT with or without ADT were likely to have been initially diagnosed with metastatic PC, and that their risk of developing CVD would be different from the surgery-only group, which was more likely to have had their PC detected by PSA screening.

Our study has important clinical implications. Our results suggest the necessity for an individualized approach to PC survivors based on primary treatment method. While surgery or RT seems to be not related to CVD risk, ADT seems to increase CVD risk. Therefore, prevention and management of CVD should be an integral part of PC survivorship care^26^, specifically following ADT. Urologists should be vigilant with regard to potential development of CVD among PC survivors, specifically when considering adjuvant or salvage ADT after surgery, and should establish links with preventive care practitioners (given most urologists do not typically undertake management of CV health in their practices).

There were several limitations to our study. First, as we used administrative data, we did not have detailed clinicopathological information, such as cancer stage, Gleason score, and recurrence. Second, we did not have information indicating whether the patients were diagnosed by PSA screening or clinical presentation, factors which may have been associated with other health behaviors related to CV risk management. Third, we do not know how the treatment decision for individual patients has been made. For example, the AS/WW group was used as reference group in comparing different CV risk among different treatment groups. The AS/WW group could be either those who could not get more invasive treatment due to frailty or those who did not want invasive treatment. Therefore, the difference in the CV risk between different treatment groups should not be interpreted as a direct effect of treatment. Fourth, the follow-up period was relatively brief (mean 4 years, maximum 10 years), and longer follow-up would be helpful to determine risks in long-term survivors. Finally, our study is based on an Asian population in which the prevalence of IHD is much lower and of stroke is much higher than that of Western populations.

## Conclusions

PC survivors were found to have a slightly lower risk of CVD compared to the general population, which was attributable to self-selection for PSA screening. This effect was most prominent in the surgery-only group, which had a markedly lower risk of CVD. In contrast, ADT was associated with increased risk of CVD. CVD risk is dependent on treatment received, and attention should be given to patients who receive ADT.

## Supplementary information

Supplementary Information.
