# Supplementary material for: Risk of Ischemic Heart Disease and Stroke in Prostate Cancer Survivors: A Nationwide Study in South Korea
Source: Sci Rep. 2020 Jun 25;10:10313. doi: 10.1038/s41598-020-67029-y (PMC7316755; doi:10.1038/s41598-020-67029-y)
Supplement: Supplementary file 1 — Supplementary Information. [file 41598_2020_67029_MOESM1_ESM.docx]

**Supplementary Table 1. Risk of ischemic heart disease, stroke, and death in prostate cancer patients compared to the matched comparison group: Competing risk analyses**

|  | **N** | **Event** | **Person-years** | **IR (per 1000)** | **Model 1** | **Model 2** | **Model 3** | **Model 4** |
| --- | --- | --- | --- | --- | --- | --- | --- | --- |
| **All participants** | | | | | | | | |
| **Ischemic heart disease** | | | | | | | | |
| Control | 200,480 | 4,022 | 764,737.6 | 5.3 | 1(Ref.) | 1(Ref.) | 1(Ref.) |  |
| Case | 48,298 | 885 | 174,673.7 | 5.1 | 0.91 (0.85, 0.98) | 0.89 (0.83, 0.95) | 0.84 (0.80, 0.90) |  |
| **Stroke** | | | | | | | | |
| Control | 200,480 | 9,028 | 754,976.4 | 12.0 | 1(Ref.) | 1(Ref.) | 1(Ref.) |  |
| Case | 48,298 | 1,980 | 172,703.5 | 11.5 | 0.91 (0.86, 0.95) | 0.88 (0.84, 0.92) | 0.85 (0.81, 0.89) |  |
| **Death** | | | | | | | | |
| Control | 200,480 | 25,926 | 771,765.1 | 33.6 | 1(Ref.) | 1(Ref.) | 1(Ref.) |  |
| Case | 48,298 | 9,658 | 176,054.8 | 54.9 | 1.64 (1.60, 1.68) | 1.60 (1.56, 1.64) | 1.61 (1.57, 1.65) |  |
| **Screening subset** | | |  |  |  |  |  |  |
| **Ischemic heart disease** | | |  |  |  |  |  |  |
| Control | 105,347 | 1,766 | 390,160.3 | 4.5 | 1(Ref.) | 1(Ref.) | 1(Ref.) | 1(Ref.) |
| Case | 29,365 | 477 | 103,946.2 | 4.6 | 0.96 (0.87, 1.07) | 0.93 (0.84, 1.03) | 0.90 (0.81, 0.99) | 0.92 (0.83, 1.02) |
| **Stroke** | | |  |  |  |  |  |  |
| Control | 105,347 | 4,002 | 385,781.1 | 10.4 | 1(Ref.) | 1(Ref.) | 1(Ref.) | 1(Ref.) |
| Case | 29,365 | 1,075 | 102,905.3 | 10.4 | 0.96 (0.89, 1.02) | 0.92 (0.86, 0.98) | 0.89 (0.84, 0.96) | 0.92 (0.86, 0.98) |
| **Death** | | |  |  |  |  |  |  |
| Control | 105,347 | 9,202 | 393,364.8 | 23.4 | 1(Ref.) | 1(Ref.) | 1(Ref.) | 1(Ref.) |
| Case | 29,365 | 4,611 | 104,694.0 | 44.0 | 1.89 (1.83, 1.96) | 1.81 (1.75, 1.88) | 1.82 (1.75, 1.89) | 1.90 (1.84, 1.98) |

Relative risks are expressed as subdistribution hazard ratio (95% confidence interval).

IR: incidence rate

Model 1: crude model

Model 2: adjusted for age

Model 3: adjusted for age, income, Charlson comorbidity index, diabetes mellitus, hypertension, and dyslipidemia

Model 4: adjusted for age, income, Charlson comorbidity index, diabetes mellitus, hypertension, dyslipidemia, smoking status, BMI, blood glucose, systolic blood pressure, and total cholesterol

**Supplementary Table 2. Risk of ischemic heart disease, stroke, and death in prostate cancer patients by treatment modality compared to the matched comparison group: Screening subset**

|  | N | Event | Duration | IR (per 1000) | Model 1 | Model 2 | Model 3 | Model 4 |
| --- | --- | --- | --- | --- | --- | --- | --- | --- |
| **Ischemic heart disease** | | |  |  |  |  |  |  |
| Control | 105,347 | 1,766 | 390,160.3 | 4.5 | 1(Ref.) | 1(Ref.) | 1(Ref.) | 1(Ref.) |
| AS/WW | 3,725 | 72 | 15,089.5 | 4.8 | 1.05 (0.83, 1.33) | 1.05 (0.83, 1.33) | 1.00 (0.79, 1.27) | 1.03 (0.82, 1.31) |
| Surgery | 11,720 | 131 | 41,475.4 | 3.2 | 0.67 (0.59, 0.84) | 0.80 (0.67, 0.96) | 0.77 (0.65, 0.92) | 0.81 (0.68, 0.97) |
| Surgery + ADT | 3,830 | 53 | 13,965.7 | 3.8 | 0.84 (0.64, 1.10) | 0.92 (0.70, 1.21) | 0.87 (0.67, 1.17) | 0.92 (0.70, 1.22) |
| RT + ADT | 885 | 9 | 1,934.3 | 4.7 | 1.06 (0.55 ,2.05) | 0.96 (0.50, 1.85) | 0.93 (0.48, 1.78) | 0.97 (0.50, 1.86) |
| ADT | 8,919 | 206 | 30,891.3 | 6.7 | 1.47 (1.28, 1.70) | 1.12 (0.97, 1.30) | 1.09 (0.94, 1.26) | 1.10 (0.95 ,1.27) |
| RT | 286 | 6 | 589.9 | 10.2 | 2.34 (1.05, 5.20 | 1.98 (0.89, 4.41) | 1.81 (0.82, 4.04) | 1.89 (0.85, 4.21) |
| **Stroke** | | |  |  |  |  |  |  |
| Control | 105,347 | 4,002 | 385,781.1 | 10.4 | 1(Ref.) | 1(Ref.) | 1(Ref.) | 1(Ref.) |
| AS/WW | 3,725 | 159 | 14,900.3 | 10.7 | 1.02 (0.87, 1.19) | 1.02 (0.87, 1.19) | 0.98 (0.84, 1.15) | 1.01 (0.86, 1.19) |
| Surgery | 11,720 | 273 | 41,227.0 | 6.6 | 0.64 (0.57, 0.73) | 0.78 (0.69, 0.88) | 0.76 (0.67, 0.86) | 0.80 (0.71, 0.91) |
| Surgery + ADT | 3,830 | 114 | 13,853.4 | 8.2 | 0.80 (0.66, 0.96) | 0.91 (0.75, 1.09) | 0.88 (0.73,1.06) | 0.92(0.77, 1.11) |
| RT + ADT | 885 | 21 | 1,918.5 | 10.9 | 1.11 (0.72, 1.70) | 0.98 (0.64, 1.51) | 0.97 (0.63,1.48) | 1.00 (0.65, 1.54) |
| ADT | 8,919 | 499 | 30,420.6 | 16.4 | 1.58 (1.44, 1.74) | 1.12 (1.02, 1.23) | 1.10 (1.00, 1.21) | 1.11 (1.01, 1.22) |
| RT | 286 | 9 | 585.5 | 15.4 | 1.55 (0.81, 2.99) | 1.27 (0.66, 2.44) | 1.19 (0.62, 2.29) | 1.26 (0.65, 2.42) |
| **Death** | | |  |  |  |  |  |  |
| Control | 105,347 | 9,202 | 393,364.8 | 23.4 | 1(Ref.) | 1(Ref.) | 1(Ref.) | 1(Ref.) |
| AS/WW | 3,725 | 738 | 15,217.9 | 48.5 | 2.02(1.87, 2.17) | 2.01 (1.87, 2.17) | 2.01 (1.86, 2.17) | 2.10 (1.95, 2.26) |
| Surgery | 11,720 | 624 | 41,705.7 | 15.0 | 0.65 (0.60, 0.70) | 0.84 (0.77, 0.91) | 0.85 (0.79, 0.92) | 0.91 (0.84, 0.99) |
| Surgery + ADT | 3,830 | 309 | 14,051.5 | 22.0 | 0.95 (0.85 ,1.06) | 1.14 (1.02, 1.28) | 1.15 (1.03, 1.29) | 1.22 (1.09, 1.37) |
| RT + ADT | 885 | 70 | 1,940.0 | 36.1 | 1.70 (1.34, 2.15) | 1.51 (1.19, 1.91) | 1.54 (1.22, 1.95) | 1.64 (1.29, 2.07) |
| ADT | 8,919 | 2,838 | 31,183.4 | 91.0 | 3.89 (3.73, 4.06) | 2.58 (2.47, 2.69) | 2.57 (2.46, 2.69) | 2.64 (2.53, 2.76) |
| RT | 286 | 32 | 595.6 | 53.7 | 2.54 (1.79, 3.59) | 2.02 (1.42, 2.85) | 2.05 (1.45, 2.90) | 2.15 (1.52, 3.05) |

IR: incidence rate

Model 1: crude model

Model 2: adjusted for age

Model 3: adjusted for age, income, Charlson comorbidity index, diabetes mellitus, hypertension, and dyslipidemia

Model 4: adjusted for age, income, Charlson comorbidity index, diabetes mellitus, hypertension, dyslipidemia, smoking status, BMI, blood glucose, systolic blood pressure, and total cholesterol

**Supplementary Table 3. Risk of ischemic heart disease, stroke, and death in prostate cancer patients by treatment modality compared to the AS/WW group: Screening subset**

|  | **N** | **Event** | **Person-year** | **IR(per 1000)** | **Model 1** | **Model 2** | **Model 3** | **Model 4** |
| --- | --- | --- | --- | --- | --- | --- | --- | --- |
| **Ischemic heart disease** | | |  |  |  |  |  |  |
| AS/WW | 3,725 | 72 | 15,089.5 | 4.8 | 1(Ref.) | 1(Ref.) | 1(Ref.) | 1(Ref.) |
| Surgery | 11,720 | 131 | 41,475.4 | 3.2 | 0.67 (0.50, 0.89) | 0.77 (0.57, 1.03) | 0.77 (0.58 ,1.04) | 0.78 (0.58, 1.05) |
| Surgery + ADT | 3,830 | 53 | 13,965.7 | 3.8 | 0.80 (0.56, 1.15) | 0.88 (0.62, 1.26) | 0.89 (0.62, 1.28) | 0.89 (0.63, 1.28) |
| RT + ADT | 885 | 9 | 1,934.3 | 4.7 | 0.10 (0.50, 2.00) | 0.90 (0.45, 1.81) | 0.91(0.45, 1.83) | 0.91 (0.45, 1.83) |
| ADT | 8,919 | 206 | 30,891.3 | 6.7 | 1.41 (1.08, 1.84) | 1.07 (0.81, 1.41) | 1.09 (0.83, 1.43) | 1.07 (0.81, 1.41) |
| RT | 286 | 6 | 589.9 | 10.2 | 2.17 (0.94, 5.01) | 1.84 (0.80, 4.26) | 1.77 (0.77, 4.08) | 1.77 (0.77, 4.10) |
| **Stroke** | | |  |  |  |  |  |  |
| AS/WW | 3,725 | 159 | 14,900.3 | 10.7 | 1(Ref.) | 1(Ref.) | 1(Ref.) | 1(Ref.) |
| Surgery | 11,720 | 273 | 41,227.0 | 6.6 | 0.63 (0.52, 0.76) | 0.73 (0.59, 0.88) | 0.73 (0.60, 0.89) | 0.74 (0.61, 0.91) |
| Surgery + ADT | 3,830 | 114 | 13,853.4 | 8.2 | 0.78 (0.61, 0.99) | 0.86 (0.67, 1.09) | 0.86 (0.68, 1.10) | 0.87 (0.68, 1.11) |
| RT + ADT | 885 | 21 | 1,918.5 | 10.9 | 1.07 (0.68, 1.69) | 0.96 (0.61, 1.52) | 0.97 (0.61, 1.53) | 0.97 (0.61 ,1.53) |
| ADT | 8,919 | 499 | 30,420.6 | 16.4 | 1.55 (1.30, 1.85) | 1.16 (0.97, 1.39) | 1.17 (0.98, 1.41) | 1.15 (0.96, 1.38) |
| RT | 286 | 9 | 585.5 | 15.4 | 1.50 (0.76, 2.94) | 1.25 (0.64, 2.46) | 1.20 (0.61, 2.36) | 1.22 (0.62, 2.39) |
| **Death** | | |  |  |  |  |  |  |
| AS/WW | 3,725 | 738 | 15,217.9 | 48.5 | 1(Ref.) | 1(Ref.) | 1(Ref.) | 1(Ref.) |
| Surgery | 11,720 | 624 | 41,705.7 | 15.0 | 0.31 (0.28, 0.34) | 0.33 (0.30, 0.37) | 0.33 (0.30, 0.37) | 0.34 (0.30, 0.38) |
| Surgery + ADT | 3,830 | 309 | 14,051.5 | 22.0 | 0.45 (0.39, 0.51) | 0.47 (0.41, 0.54) | 0.47 (0.41, 0.54) | 0.47 (0.41, 0.54) |
| RT + ADT | 885 | 70 | 1,940.0 | 36.1 | 0.72 (0.60, 0.91) | 0.66 (0.52, 0.85) | 0.67 (0.53, 0.86) | 0.68 (0.53, 0.87) |
| ADT | 8,919 | 2838 | 31,183.4 | 91.0 | 1.86 (1.72, 2.02) | 1.55 (1.43, 1.68) | 1.53 (1.41, 1.66) | 1.50 (1.38, 1.63) |
| RT | 286 | 32 | 595.6 | 53.7 | 1.07 (0.75, 1.52) | 0.95 (0.67, 1.35) | 0.97 (0.68, 1.38) | 0.97 (0.68, 1.38) |

IR: incidence rate

Model 1: crude model

Model 2: adjusted for age

Model 3: adjusted for age, income, Charlson comorbidity index, diabetes mellitus, hypertension, and dyslipidemia

Model 4: adjusted for age, income, Charlson comorbidity index, diabetes mellitus, hypertension, dyslipidemia, smoking status, BMI, blood glucose, systolic blood pressure, and total cholesterol
